# Why checking model assumptions using null hypothesis significance tests does not suffice: A plea for plausibility

**DOI:** 10.3758/s13423-018-1447-4

**Published:** 2018-02-23

**Authors:** Jesper Tijmstra

**Affiliations:** Department of Methodology and Statistics, Faculty of Social Sciences, Tilburg University, PO Box 90153, 5000 LE Tilburg, The Netherlands

**Keywords:** Statistical inference, Bayesian statistics, Belief updating, Statistics

## Abstract

This article explores whether the null hypothesis significance testing (NHST) framework provides a sufficient basis for the evaluation of statistical model assumptions. It is argued that while NHST-based tests can provide some degree of confirmation for the model assumption that is evaluated—formulated as the null hypothesis—these tests do not inform us of the degree of support that the data provide for the null hypothesis and to what extent the null hypothesis should be considered to be plausible after having taken the data into account. Addressing the prior plausibility of the model assumption is unavoidable if the goal is to determine how plausible it is that the model assumption holds. Without assessing the prior plausibility of the model assumptions, it remains fully uncertain whether the model of interest gives an adequate description of the data and thus whether it can be considered valid for the application at hand. Although addressing the prior plausibility is difficult, ignoring the prior plausibility is not an option if we want to claim that the inferences of our statistical model can be relied upon.

## Introduction

One of the core objectives of the social sciences is to critically evaluate its theories on the basis of empirical observations. Bridging this gap between data and theory is achieved through statistical modeling: Only if a statistical model is specified can the data be brought to bear upon the scientific theory. Without making assumptions about the statistical model, no conclusions can be drawn about the hypotheses of substantive interest. While in practice these statistical model assumptions may often be glossed over, establishing these assumptions to be plausible is crucial for establishing the validity of the inferences: Only if the statistical model is specified (approximately) correctly can inferences about hypotheses of interest be relied upon. Hence, critically evaluating the statistical model assumptions is of crucial importance for scientific enquiry. These statistical model assumptions can themselves be investigated using statistical methods, many of which make use of null-hypothesis significance tests (NHST).

As the statement of the American Statistical Association exemplifies (Wasserstein & Lazar, 2016), recently much attention in psychology and related fields has been devoted to problems that arise when NHST is employed to evaluate substantive hypotheses. Criticisms of NHST are usually two-fold. Firstly, it is noted that in practice NHST is often abused to draw inferences that are not warranted by the procedure (Meehl, 1978; Cohen, 1994; Gigerenzer & Murray, 1987; Gigerenzer, 1993). For instance, it is noted that NHST is not actually used to critically test the substantive hypothesis of interest (Cohen, 1994), that practitioners often conclude that a significant result indicates that the null hypothesis is false and the alternative hypothesis is true or likely to be true (Gigerenzer, 2004), that a *p* value is taken to be the probability that the observed result was due to chance (rather than a probability conditional on the null hypothesis being true, Cohen, 1994; Wagenmakers, 2007), and that a significant result is taken to indicate that the finding is likely to replicate (Gigerenzer, 2000). The second criticism is that the types of inferences that one can draw on the basis of NHST are of limited use for the evaluation of scientific theories (Gigerenzer & Murray, 1987; Gigerenzer, 1993, 2004), with alternatives such as using confidence intervals (Cumming & Finch, 2005; Fidler & Loftus, 2009; Cumming, 2014) or Bayesian approaches (Wagenmakers, 2007) being proposed as more informative and more appropriate tools for statistical inference. These criticisms have been unanimous in their rejection of the use of (solely) NHST-based methods for evaluating substantive hypotheses and have had an important impact on both recommended and actual statistical practices, although the uncritical and inappropriate use of NHST unfortunately still appears to be quite common in practice.

The attention in the discussion of the (in)adequacy of NHST has focused almost exclusively on the use of NHST for the evaluation of substantive hypotheses. Importantly, standard methods for assessing whether statistical model assumptions hold often rely on NHST as well (e.g., Tabachnick & Fidell, 2001; Field, 2009), and are even employed when the substantive analyses do not use NHST (e.g., item response theory; Lord, 1980). These NHST procedures formulate the model assumption as a statistical hypothesis, but—unlike the statistical hypothesis derived from the substantive hypothesis—treat this hypothesis as a null hypothesis to be falsified. It may also be noted here that despite users usually being interested in determining whether the model assumption at least approximately holds, the null hypothesis that is tested specifies that the model assumption holds *exactly*, something that often may not be plausible a priori. Given the differences in the way NHST deals with model assumptions compared to substantive hypotheses, given the different purpose of assessing model assumptions (i.e., evaluating the adequacy of the statistical model), and given the common usage of NHST for evaluating model assumptions, it is important to specifically consider the use of NHST for the evaluation of model assumptions and the unique issues that arise in that context.

This paper explores whether NHST-based approaches succeed in providing sufficient information to determine how plausible it is that a model assumption holds, and whether a model assumption is plausible enough to be accepted. The paper starts out with a short motivating example, aimed at clarifying the specific issues that arise when model assumptions are evaluated using null hypothesis tests. In “The NHST framework”, the background of the NHST framework is discussed, as well as the specific issues that arise when it is applied to evaluate model assumptions. “Confirmation of model assumptions using NHST” explores the extent to which a null hypothesis significance test can provide confirmation for the model assumption it evaluates. “Revisiting the motivating example” briefly returns to the motivating example, where it is illustrated how Bayesian methods that do not rely on NHST may provide a more informative and appropriate alternative for evaluating model assumptions.

## A motivating example

Consider a researcher who wants to find out whether it makes sense to develop different types of educational material for boys and for girls, as (we assume) is predicted by a particular theory. Specifically, the researcher is interested in children’s speed of spatial reasoning. She constructs a set of ten items to measure this ability and for each respondent records the total response time.

Assume that the researcher plans on using Student’s independent samples *t* test to compare the average response speed of boys and girls. Before she can interpret the results of this *t* test, she has to make sure that the assumptions are met. In this case, she has to check whether both the response times of the boys and the response times of the girls are independently and identically distributed, whether the distributions are Gaussian, and whether the variances of these distributions are the same (Student, 1908; Field, 2009). The researcher is aware of the unequal variances *t* test (Welch, 1947) (i.e., a different statistical model), but because this procedure has lower power, she prefers to use Student’s.

For simplicity, consider only the assumption of equal variances. In this case, Levene’s test (Levene, 1960) can be used to evaluate the null hypothesis $H_{0}: \sigma ^{2}_{boys} = \sigma ^{2}_{girls}$. Assume that Levene’s test yields a *p* value of 0.060, which exceeds the level of significance (which for Levene’s test is usually set to 0.05 or 0.01, Field, 2009).^1^ The researcher concludes that there is no reason to worry about a violation of the assumption of equal variances, and proceeds to apply Student’s *t* test (using *α* = 0.05). The *t* test shows that boys are significantly faster than girls in solving the spatial reasoning items (*p* = 0.044). The researcher notes that her statistical software also produces the output for the *t* test for groups with unequal variances (Welch, 1947), which yields *p* = 0.095. However, since Levene’s test did not indicate a violation of the assumption of equal variances, the researcher feels justified in ignoring the results of the Welch test and sticks to her conclusion that there is a significant difference between the two groups.

The present example is meant to exemplify how statistical model assumptions are commonly dealt with in practice (see also Gigerenzer, 2004). Important questions can be asked about the justification of the inferences made in this example, and in cases like this in general. Should the researcher be worried about her model assumption if she has background knowledge that boys commonly show a larger variance in cognitive processing speed than girls? Can she safely conclude that the variances are indeed equal in the population and that the model assumption holds, or that this is at least very likely? And if there remains uncertainty about the plausibility of this model assumption, how should this uncertainty influence her statistical inferences?

Questions like these are not restricted to the application of *t* tests, but apply equally strongly to all areas where statistical models are used to make inferences, and includes areas where the option of simply using a less restrictive model (e.g., using Welch’s *t* test rather than Student’s) is not readily available. Since statistical inferences based on these models often form the basis for updating our scientific beliefs as well as for taking action, determining whether we can rely on inferences that are made about model assumptions using NHST is of both theoretical and practical importance.

## The NHST framework

### Background of the NHST framework

The basis of the NHST framework goes back to the statistical paradigm founded by Fisher in the 1930s (Fisher, 1930, 1955, 1956, 1960; Hacking, 1976; Gigerenzer, 1993), as well as the statistical paradigm founded by Neyman and Pearson in that same period (Neyman, 1937, 1957; Pearson, 1955; Neyman & Pearson, 1967; Hacking, 1976; Gigerenzer, 1993). Whereas Neyman and Pearson proposed to contrast two hypotheses that are in principle on equal footing, Fisher’s approach focuses on evaluating the fit of a single hypothesis to the data, and has a strong focus on falsification. Starting from the 1950s, elements from both approaches were incorporated in the hybrid NHST framework as it exists today in the social and behavioral sciences (Gigerenzer & Murray, 1987; Gigerenzer, Swijtink, Porter, Daston, Beatty, & Krüger, 1989; Gigerenzer, 1993; Lehmann, 2006): While this framework proposes to evaluate a null hypothesis in contrast to an alternative hypothesis—in line with Neyman and Pearson—the focus lies on attempting to reject the null hypothesis, in line with Fisher’s methodology. Thus, despite the important differences that existed between the paradigms of Fisher and that of Neyman and Pearson (Gigerenzer, 1993), the current framework constitutes a hybrid form of the two paradigms (Gigerenzer et al., 1989; Gigerenzer, 1993; Hubbard & Bayarri, 2003).

In line with Fisher, in NHST, the evaluation of the null hypothesis is done solely based on the *p* value: the probability of observing an outcome under the null hypothesis that is at least as extreme as the outcome that was observed. If the *p* value falls below a preset level of significance *α*, it is concluded that the data are unlikely to be observed under the null hypothesis (in line with Fisher), in which case the null hypothesis is rejected and the alternative hypothesis is accepted (in line with Neyman and Pearson). If *p* ≥ *α*, the null hypothesis is retained, but no conclusions are drawn about the truth of the null hypothesis (Fisher, 1955).

Because the NHST framework explicitly does not address the probability that the null hypothesis is true (Edwards, Lindman & Savage, 1963), NHST remains silent about the effect that a failure to reject the null hypothesis should have on our assessment of the plausibility of the null hypothesis—the extent or degree to which we believe that it is credible or likely to be true. Similar to the way in which Popper suggests to evaluate theories (Popper, 1959), null hypotheses are simply considered hypotheses that have not yet successfully been rejected, but should not be considered to be likely to be true.

### Differences between evaluating substantive hypotheses and model assumptions

Many authors have criticized the use of NHST for the evaluation of substantive hypotheses (see for example Meehl, 1978; Cohen, 1994; Gigerenzer, 2004; Wagenmakers, 2007). However, the use of NHST for the evaluation of model assumptions differs in at least three important ways from the use of NHST for evaluating substantive theories, making it important to specifically consider the merits and demerits of employing NHST in the context of evaluating model assumptions.

One important difference lies in the type of inference that is desired. In the context of evaluating scientific theories, one can argue that conclusions about substantive hypotheses are always *provisional*, and that we should refrain from drawing any strong conclusions on the basis of a single hypothesis test, requiring instead that the results are to be consistently replicable. In this context, one can argue (Mayo, 1996) that the only probabilities that are relevant are the error probabilities of the procedure (in the case of NHST the Type I and Type II error), and one can in principle refrain from taking action on the basis of finite, and hence principally inconclusive, evidence. Unfortunately, this appeal to the long-run success of the procedure is not available when evaluating model assumptions, since in that context we always need to make a *decision* about whether *in this particular instance* we will proceed to apply the statistical model (i.e., acting as though its assumptions have been met).

A second important difference lies in the fact that when evaluating model assumptions using NHST, the null hypothesis is actually the hypothesis of interest, rather than a ‘no effect’ or ‘nil’ hypothesis (Cohen, 1994) that the researcher hopes to reject in favor of the proposed alternative. Thus, while substantive hypotheses are only evaluated indirectly by attempting to reject a null hypothesis, when model assumptions are concerned, researchers are testing the relevant hypothesis ‘directly’, sticking (at least at first glance) more closely to the idea that one should aim to falsify one’s hypotheses and expose them to a critical test (see e.g., Mayo, 1996). In this sense, the use of NHST for evaluating model assumptions may escape the criticism (Cohen, 1994) that the hypothesis of interest is not the hypothesis that is actually critically tested.

A third difference concerns the desired control over NHST’s two main error probabilities: the probability of incorrectly rejecting the null hypothesis when it is true (Type I error), and the probability of failing to reject it when it is false (Type II error). By testing the hypothesis of interest directly, the use of NHST for evaluating model assumptions implies an important shift in the relative importance of Type I and Type II errors compared to its use for the evaluation of substantive hypotheses. In this latter setting, the standard approach to using NHST is to fix the level of significance, ensuring control over the Type I error rate, and only then (if at all) consider the power of the procedure to detect meaningful deviations from this null hypothesis. However, when model assumptions are concerned, Type II errors are arguably much more harmful than Type I errors, as they result in the unwarranted use of statistical procedures and in incorrect inferences about the substantive hypotheses (e.g., due to inflated Type I error rates, overly small confidence intervals, or parameter bias). Thus, the standard practice of mainly focusing on controlling the Type I error rate by selecting an acceptably low level of significance *α* does not appear to translate well to the evaluation of model assumptions, since this leaves the more important Type II error rate uncontrolled and dependent on factors such as sample size that should arguably not affect the severity with which we critically evaluate our assumptions. While controlling the Type I error rate may often be straightforward, controlling the Type II error rate will be problematic for researchers who do not assess the plausibility of model assumptions and the size of possible violations, as will be shown in “Assessing the plausibility of *H*_0_ using a null hypothesis test”.

### Using null hypothesis tests to evaluate model assumptions

The standard way in which NHST is used to evaluate a model assumption is by formulating that assumption as a simple null hypothesis, stating that, for example, a set of parameters (e.g., group variances) are exactly the same. While less common, one could also consider using the NHST framework to assess whether a model assumption is approximately satisfied; that is, whether it falls within the bounds of what is considered acceptable for the model when we take the robustness of the model against violations of that assumption into account. Under such an approach, one would consider a composite null hypothesis that states that the differences between those parameters are not too large (e.g., that none of the group variances differ by more than a factor 10) for the model inferences to be seriously affected (see also Berger & Delampady, 1987; Hoijtink, 2012). While the statistical hypothesis that is tested would differ, the motivation for testing that hypothesis using NHST would be the same: to determine whether inferences based on the model can be trusted. Because of this similarity, the issues concerning the use of NHST for evaluating model assumptions discussed in this paper apply equally to the use of standard null hypothesis tests and approximate null hypothesis tests.^2^ Since practically all standard use of NHST for evaluating model assumptions makes use of simple null hypotheses these shall be the focus of most of the article, but the relevance of using approximate null hypotheses is revisited at the end of “Assessing the plausibility of *H*_0_ using a null hypothesis test”.

The difficulty lies in the fact that the NHST framework normally informs us that a nonrejection of the null hypothesis does not confirm the null hypothesis. This strict and Fisherian application of the NHST framework (here called ‘strict approach’) only informs us whether the model should be considered to be inappropriate and not whether we can assume that it is correct. While this strict position may be theoretically defensible, its implications are highly restrictive and do not match scientific practice. That is, using only NHST, no model assumption would ever receive any degree of confirmation from the data, and their plausibility would always remain completely uncertain. Since claiming that we never have any evidence in favor of statistical model assumptions would make all statistical inference arbitrary, it is assumed in the remainder of the paper that the application of the NHST framework to model assumptions only makes sense if it allows for some form of confirmation of the null hypothesis, and hence that the strict approach cannot be defended in practice.

One way to avoid the implication that we cannot put any faith in our statistical models is to change the implication of a nonrejection of *H*_0_: Instead of retaining it, we could decide to accept it. This ‘confirmatory approach’ seems to be implicitly embraced in practice (and in the motivating example), where researchers check their assumptions and in the absence of falsification proceed as though the assumption has been confirmed. It can also be thought to be in line with the decision-oriented methodology of Neyman and Pearson, where after performing a statistical test one of the two hypotheses is accepted. However, for this confirmatory approach to be defensible, it has to be argued that a nonrejection of *H*_0_ should provide us with sufficient reason to accept *H*_0_, which will be explored in the next section.

Another possible response to the problems of the strict approach to NHST is to abandon the idea that a dichotomous decision needs to be made about the model assumption based on the outcome of the null hypothesis test. Instead, one could decide to always continue using the statistical model, while taking into consideration that a model assumption for which a significant result was obtained may be less plausible than one for which no significant result was obtained. This would mean treating the significance of the test statistic as a dichotomous measure that provides us with some degree of support in favor (i.e., nonsignificant) or against (i.e., significant) the model assumption, which is evidence that we should take into account when determining the extent to which we can rely on inferences based on the model. The feasibility of this ‘evidential approach’ to NHST is also considered in the next section.

## Confirmation of model assumptions using NHST

The previous section concluded with two possible adaptations of the NHST framework that could potentially make NHST suitable for the evaluation of model assumptions. However, the possible success of both approaches hinges on whether NHST is able to provide the user with sufficient information to assess the plausibility of the model assumption. This section explores whether this is the case, for which the concept of plausibility will first need to be further defined.

### Prior and posterior plausibility of the model assumption

Let us formalize the notion of plausibility by requiring it to take on a value that can range from 0 (completely implausible or certainly wrong) to 1 (completely plausible or certainly right) (see also Jaynes, 1968, 2003). This value represents the degree of plausibility that is assigned to a proposition, for example the proposition ‘*H*_0_ is true’.

Since model assumptions are arguably either true or false, if we had complete information, there would be no uncertainty about the model assumptions and we would assign a value of either 0 or 1 to the plausibility of a model assumption being true. However, researchers are forced to assess the plausibility of the assumption using incomplete information, and their assessment of the plausibility depends on the limited information that they have and the way in which they evaluate this information. Thus, when we speak of the plausibility of a model assumption, it will always be conditional on the person that is doing the evaluation and the information that she has.

Denote the plausibility of a hypothesis *H*_0_ as it is assessed by a rational and coherent person *j* by *P*_*j*_(*H*_0_) (see also Jaynes, 2003). Such rational and coherent persons may not actually exist, but can serve as idealizations for the way in which we should revise our beliefs in the face of new evidence. Thus, *P*_*j*_(*H*_0_) represents the degree to which person *j* believes in the proposition ‘*H*_0_ is true’, and it therefore informs us to what extent person *j* thinks that it is probable that *H*_0_ is true. In line with the Bayesian literature on statistics and epistemology, this ‘degree of belief’ could also be called the ‘subjective probability’ or ‘personal probability’ that a person assigns to the truth of a proposition (see Savage, 1972; Howson & Urbach, 1989; Earman, 1992; Suppes, 2007). As a way of quantifying this degree of belief, we could imagine asking this person how many cents she would be willing to bet on the claim that *H*_0_ is true if she will receive 1 dollar in the case that *H*_0_ is indeed true (Ramsey, 1926; Gillies, 2000).

It is important to emphasize that this degree of belief need not be arbitrary, in the sense of depending on the subjective whims or preferences of the person (Jeffreys, 1961; Howson & Urbach, 1989; Lee & Wagenmakers, 2005). Rather, we can demand that the belief is rationally constructed on the basis of the set of information that is available to the person and a set of rules of reasoning (Jaynes, 1968, 2003). The idea would be to use methods to elicit the relevant information that a person may have and to translate this information through a carefully designed and objective set of operations into an assessment of the prior plausibility (see also Kadane & Wolfson, 1998; O’Hagan, 1998; O’Hagan et al., 2006). This assessment would only be subjective in the sense that persons might differ in the information that is available to them, and we may require that persons with the same information available to them should reach the same assessment of prior plausibility, making the assessment itself objective.

Let us assume that person *j* has obtained a data set ***X***—the data to which she hopes to apply the model—and that she wants to determine how plausible it is that *H*_0_ holds after having taken the data into consideration. Let us call her prior assessment *P*_*j*_(*H*_0_) of the plausibility of *H*_0_ before considering the data ***X*** the *prior plausibility*. Since person *j* wants to determine whether she should trust inferences based on the model, she wants to make use of the information in the data to potentially improve her assessment of the plausibility of *H*_0_. Thus, to make a better assessment of how plausible *H*_0_ is, she wants to update her prior belief based on the information in the data. Let us call this assessment of the plausibility that has been updated based on the data ***X*** person *j*’s posterior plausibility, which we denote by *P*_*j*_(*H*_0_|***X***).

### Relevance of prior knowledge about the model assumption

Since both the confirmatory and the evidential approach to NHST posit that our evaluation of the plausibility of *H*_0_ should be based only on the data (see also Trafimow, 2003), they tell us that our informed prior beliefs about the possible truth of *H*_0_ should be completely overridden by the data. The idea is that this way the influence of possible subjective considerations is minimized (see also Mayo, 1996). Proponents of the NHST framework cannot allow the prior assessment of the plausibility of *H*_0_ to influence the conclusions that are drawn about the plausibility of *H*_0_ without abandoning the idea that the *p* value contains all the relevant information about the plausibility of *H*_0_. Hence, it will be assumed that if person *j* follows NHST-based guidelines in assessing the plausibility of *H*_0_, *P*_*j*_(*H*_0_|***X***) will not depend on *P*_*j*_(*H*_0_), but solely depends on the *p* value.

However, there are clear cases where our assessment of the plausibility of *H*_0_*should* depend on our prior knowledge if we are to be consistent. If for some reason we already know the truth or falsehood of *H*_0_, then basing our assessment of the plausibility purely on the result of a null hypothesis test—with the possibility of a Type I and Type II error, respectively—can only make our assessment of the plausibility of *H*_0_ worse, not better. When we know in advance that a model assumption is wrong, failing to reject it should not in any way influence our assessment of the assumption.

More generally, one can conclude that the less plausible a null hypothesis is on the basis of the background information, the more hesitant we should be to consider it to be plausible if it fails to be rejected. In the context of the motivating example, the researcher may be aware of previous research indicating that boys generally show greater variance in cognitive processing speed than girls. This could give her strong reasons to suspect that boys will also show greater variance on her particular measure than girls. This background information would then be incorporated in her assessment of the plausibility of the assumption of equal variance before she considers the data. In this case, the researcher would assign low prior plausibility (e.g., *P*_*j*_(*H*_0_) = .1 or even *P*_*j*_(*H*_0_) = .01) to the assumption of equal variances. The data may subsequently provide us with relevant information about the model assumption, but this should influence our assessment of the plausibility of *H*_0_ in a way that is consistent with our prior assessment of the plausibility of *H*_0_. That is, *P*_*j*_(*H*_0_|**X**) should depend on *P*_*j*_(*H*_0_).

### Assessing the plausibility of *H*_0_ using a null hypothesis test

To examine how NHST may help to evaluate the plausibility of a statistical model assumption, let us further examine the hypothetical case of researcher *j* who wants to apply a statistical model to a data set ***X***, and who wants to evaluate one of the assumptions defining that model. In line with standard practice, let us assume that the model assumption that she evaluates is formulated as a simple null hypothesis, specifying that a parameter has a specific value. For example, this null hypothesis could correspond to the assumption of equal variances that was discussed in the motivating example, in which case *H*_0_ : *δ* = 1, where $\delta = \sigma ^{2}_{boys} / \sigma ^{2}_{girls}$.

The researcher has some prior beliefs about the plausibility of this assumption, based on the background information that she has about the particular situation she is dealing with. Since research never takes place in complete isolation from all previous research or substantive theory, the researcher will always have some background knowledge that is relevant for the particular context that she is in. If the researcher assigns either a probability of 0 or 1 to the model assumption being true before observing the data, she will consider statistically testing this hypothesis to be redundant. Thus, if researcher *j* tests *H*_0_, we have to assume that

1 $$ 0 < P_{j}(H_{0}) < 1. $$

Let us also assume that the researcher applies a null hypothesis test to the data ***X***, and that she contrasts *H*_0_ with an alternative simple hypothesis *H*_*i*_ (e.g., specifying a specific non-unity ratio for the variances). This procedure results either in a significant or a nonsignificant test statistic. For now, we will assume that the researcher follows the guidelines of the confirmatory approach to NHST. Thus, a significant test statistic results in the researcher rejecting *H*_0_—the event of which is denoted by *R*—and a nonsignificant value means that *H*_0_ is accepted—denoted by ¬*R*.

For the test statistic to provide some form of justification for accepting (or rejecting) *H*_0_ over *H*_*i*_, it must also be the case that

2 $$ P(R|H_{0}) < P(R|H_{i}). $$

From Eq. 2 it follows that

3 $$ P(\neg R|H_{0}) = 1 - P(R|H_{0}) > P(\neg R|H_{i}) = 1 - P(R|H_{i}). $$

Thus, a failure to reject *H*_0_ is more likely under *H*_0_ than under *H*_*i*_.

For convenience, let us assume that Eq. 2 holds for all possible simple alternatives of *H*_0_ (which are all mutually exclusive and which as a set together with *H*_0_ are exhaustive),

4 $$ P(R|H_{0}) < P(R|H_{i}), \text{for all \textit{i}}. $$

Equation 4 generally holds for NHST-based tests for model assumptions, such as Levene’s test for equality of variances (Levene, 1960). Let us denote the composite hypothesis that is the complement of *H*_0_ by ¬*H*_0_. Because the complement incorporates all possible alternatives to *H*_0_, ¬*H*_0_ is also known as the ‘catch-all’ hypothesis (Fitelson, 2006, 2007). For the evaluation of model assumptions using NHST, the alternative hypothesis needs to correspond to the catch-all hypothesis, since the two hypotheses together should be exhaustive if we are to assess the plausibility of the assumption.

Our assessment of the probability of obtaining a nonsignificant test statistic under the catch-all hypothesis depends on how plausible we consider each of the possible alternatives to *H*_0_ to be. That is,

5 $$ \beta_{j} = P_{j}(\neg R|\neg H_{0}) = \frac{{\sum}_{i}P(\neg R|H_{i})P_{j}(H_{i})}{{\sum}_{i}P_{j}(H_{i})}, $$

where *β*_*j*_ denotes person *j*’s assessment of the probability of a Type II error. Thus, *β*_*j*_ depends on the person that evaluates it, since *P*(*R*|*H*_*i*_) will differ for different *H*_*i*_s and persons may differ with respect to their values for each *P*_*j*_(*H*_*i*_). The power to detect a violation of the model assumption under the catch-all hypothesis thus cannot be assessed without considering the prior plausibility of each of the possible alternatives to *H*_0_.^3^ In the context of our motivating example, our assessment of the power of the test depends on what values for $\sigma ^{2}_{boys}$ and $\sigma ^{2}_{girls}$ we consider to be plausible. If we expect a large difference between the two variances, we would expect the testing procedure to have a higher power to detect these differences than if we expect a small difference.

Equation 4 implies that

6 $$ \alpha = P(R|H_{0}) < 1 - \beta_{j}. $$

Equation 6 informs us that the power of the test to detect a violation of the model assumption is larger than the probability of a Type I error given the truth of *H*_0_. From Eq. 6 it also follows that

7 $$ P(\neg R|H_{0}) = 1 - P(R|H_{0}) > 1 - P_{j}(R|\neg H_{0}) = P_{j}(\neg R|\neg H_{0}). $$

Equation 7 informs us that a nonrejection is more likely under *H*_0_ than under ¬*H*_0_. Thus, obtaining a nonsignificant test statistic should increase person *j*’s assessment of the plausibility of *H*_0_,

8 $$ P_{j}(H_{0}|\neg R) > P_{j}(H_{0}). $$

Hence, a null hypothesis test can indeed provide some degree of confirmation for the model assumption it evaluates. However, based on Eq. 8 alone, we do not know *how plausibleH*_0_ is after a failure to reject it, nor do we know *how much more plausible* it has become due to this nonrejection.

The degree to which *H*_0_ has become more plausible after having obtained a nonsignificant test statistic can be determined (see also Kass & Raftery, 1995; Trafimow, 2003) by means of

9 $$ \frac{P_{j}(H_{0}|\neg R)}{P_{j}(\neg H_{0}|\neg R)} \,=\, \frac{P_{j}(H_{0})}{P_{j}(\neg H_{0})}\!\frac{P(\neg R|H_{0})}{P_{j}(\neg R|\neg H_{0})} \,=\, \frac{P_{j}(H_{0})}{P_{j}(\neg H_{0})}\!\frac{1 - \alpha}{\beta_{j}}. $$

That is, the odds of *H*_0_ versus ¬*H*_0_ increase by a factor $\frac {1 - \alpha }{\beta _{j}}$ after having obtained a nonsignificant test statistic,^4^ which depends on our assessment of the power of the procedure. To determine how plausible *H*_0_ is after having obtained a nonsignificant result, we thus cannot avoid relying on a subjective assessment of the power of the test based on what we consider to be plausible alternatives to *H*_0_.

By combining Eq. 9 with the fact that *P*_*j*_(*H*_0_) = 1 − *P*_*j*_(¬*H*_0_), we can obtain the plausibility of *H*_0_ after having observed a nonsignificant result through

10 $$\begin{array}{@{}rcl@{}} P_{j}\!(H_{0}|\neg R) & \,=\, & \frac{P_{j}(H_{0})P(\neg R|H_{0})}{P_{j}(H_{0})P(\neg R|H_{0}) + P_{j}(\neg H_{0})P_{j}(\neg R|\neg H_{0})}\\ & \,=\,& P_{j}(H_{0})\frac{1 - \alpha}{\beta_{j} + P_{j}(H_{0})(1 - \alpha - \beta_{j})}. \end{array} $$

Equation 10 shows that our conclusion about the plausibility of *H*_0_ should depend on our prior assessment of its plausibility. It also shows that the degree to which *H*_0_ has become more plausible depends on our assessment of the power of the test, which Eq. 5 shows depends on the prior plausibility of the different specific alternatives to *H*_0_. Thus, it is not possible to assess the degree to which the data support *H*_0_ through NHST alone, and the evidential approach to NHST cannot succeed if it does not take the prior plausibility into account.

Equation 10 also illustrates why the confirmatory approach to NHST cannot provide us with defensible guidelines for accepting or rejecting *H*_0_. Since the confirmatory approach does not take the prior plausibility of *H*_0_ into account, it has to make a decision about the plausibility of *H*_0_ based on the *p* value alone (Trafimow, 2003). However, the *p* value only tells us whether the data are consistent with the assumption being true, not whether this assumption is actually likely to be true. That is, a *p* value only informs us of the probability of obtaining data at least as extreme as the data that were actually obtained conditional on the truth of the null hypothesis, and it does not represent the probability of that hypothesis being true given the data (Wagenmakers, 2007).

The fact that in practice the *p* value is often misinterpreted as the probability that the null hypothesis is true (see e.g., Guilford, 1978; Gigerenzer, 1993; Nickerson, 2000; Wagenmakers & Grunwald, 2005; Wagenmakers, 2007) already suggests that it is this probability that researchers are often interested in Gigerenzer (1993). However, because they do not address the prior plausibility of the assumption, both the confirmatory and the evidential approach to NHST are unable to inform the user how plausible it is that the assumption is true after having taken the data into consideration.

Proponents of NHST might argue that we still should avoid the subjective influence introduced by including the prior plausibility in our assessment of model assumptions, and that an objective decision rule based on the confirmatory approach is still acceptable. They might state that we simply have to accept uncertainty about our decision about the model assumption: If we repeatedly use NHST to evaluate model assumptions, we will be wrong in a certain proportion of times, and this is something that simply cannot be avoided. But the problem is that the proportion of times we can expect to be wrong if we simply accept *H*_0_ when the test statistic is not significant also depends on the prior probability of *H*_0_, as Eq. 10 shows. If our model assumption cannot possibly be true, all failures to reject *H*_0_ are Type II errors, and the decision to accept *H*_0_ will be wrong 100% of the time. Thus, this uncertainty about the proportion of times that an accepted null hypothesis is in fact wrong cannot at all be assessed without also assessing the prior plausibility. However, it seems plausible that in the context of evaluating model assumptions. it is exactly this error rate (rather than the Type I and Type II error rates) that is of most importance, as it determines the proportion of times that we end up using an inappropriate statistical model if we would rely solely on NHST.

To illustrate the risks of adopting the confirmatory approach to NHST, we can consider the proportion of times that a person following this approach can expect a conclusion that *H*_0_ is true to be incorrect. Adopting the confirmatory approach to NHST would mean accepting *H*_0_ whenever *p* ≥ *α*, regardless of its posterior plausibility. If we accept a hypothesis for which we would assess the posterior plausibility to be .70, we should expect such a decision to be wrong 30% of the time (provided the prior plausibility fully matched our prior beliefs). Thus, Eq. 10 can be used to determine the proportion of acceptances of *H*_0_ that one can expect to be incorrect, for a given assessment of the power and prior plausibility.

The impact of the prior plausibility on the proportion of incorrect acceptances of *H*_0_ based on a null hypothesis test (*α* = .05) is illustrated in Table 1. These results show that even with an assessed power (1 − *β*_*j*_) as high as .90, person *j* can determine that she should expect to incorrectly accept *H*_0_ in about 49% of times for hypotheses that she a priori considers to be implausible (*P*_*j*_(*H*_0_) = .1), or even 91% of times if she considers hypotheses for which *P*_*j*_(*H*_0_) = .01. Thus, while power analysis is important in evaluating the support that the model assumption receives, Table 1 illustrates that a high power (or a claim about severity, Mayo, 1996) alone is not sufficient to result in convincing claims that the assumption is plausible enough to be accepted (barring hypothetical cases where the power is 1). Without assessing the prior plausibility, the plausibility of the assumption after having observed the data can have any value between 0 and 1 regardless of the *p* value that is obtained.

**Table 1:** Proportion of acceptances of *H*_0_ based on a null hypothesis test that person *j* should expect to be wrong, for varying levels of power and prior plausibility (*α* = .05)

*P*_*j*_(*H*_0_)	1 − *β*_*j*_				
	.20	.50	.80	.90	.99
.01	.99	.98	.95	.91	.51
.10	.88	.83	.65	.49	.09
.20	.77	.68	.46	.30	.04
.50	.46	.34	.17	.10	.01
.80	.17	.12	.05	.03	.00
.90	.09	.06	.02	.01	.00

It is important here to emphasize that often model assumptions are chosen not because they are deemed plausible, but because of their mathematical convenience or usefulness to develop a statistical model. If there is no substantive theory that backs up these model assumptions with convincing arguments, there is little reason to assume that the model assumptions actually hold exactly, and assigning a potentially very low (possibly 0) prior plausibility to these assumptions may be the only reasonable response. Consequently, testing such a priori implausible exact null hypotheses in the hopes of finding enough support to accept them may often be hopeless, since “[e]ssentially, all models are wrong” (Box and Draper, 1987, p. 424). Our models try to simplify reality with the goal of representing it in a convenient and useful way, but because of this simplification those models often cannot completely capture the vast complexity of the reality they try to represent. As such, the idea that they are completely correct may in many cases be highly implausible.

Luckily, a model does not need to be exactly correct for its inferences to be useful. Many models are robust to small or even large violations of their assumptions, meaning that inferences made using the model might still be (approximately) correct if the violations of the assumptions are not too severe. This suggests that what researchers should be after in evaluating model assumptions is not confirming an a priori dismissible exact null hypothesis, but rather an approximate null hypothesis (Berger & Sellke, 1987; Hoijtink, 2012) that specifies an admissible range of values rather than point values for the parameter(s) that relate to the model assumption. Such ‘robust’ approximate null hypotheses might have a much higher prior plausibility, making the effort to determine whether they should be accepted more reasonable. Such hypotheses are also more in line with what researchers are interested in: figuring out whether the model assumption is not violated beyond an acceptable limit. But regardless of whether we formulate our model assumptions in the form of an exact null hypothesis or an approximate null hypothesis, we want to assess the plausibility of the assumptions that we have to make. Hence, regardless of the precise specification of the null hypothesis, the prior plausibility of that assumption needs to be assessed.

## Revisiting the motivating example

Since relying on NHST for the evaluation of the assumption of equal variances will not provide her with a sufficient assessment of the plausibility of that assumption, our researcher could decide to make use of statistical methods that do provide her with the information she needs. One useful and accessible method for evaluating this assumption has been proposed by Boing-Messing, van Assen, Hofman, Hoijtink, & Mulder (2017), who make use of a Bayesian statistical framework to evaluate hypotheses about group variances. Their method allows users to contrast any number of hypotheses about the relevant group variances. For each pair of hypotheses, their procedure calculates a Bayes factor, which captures the degree of support that the data provide in favor of one hypothesis over the other (Kass & Raftery, 1995). The Bayes factor can range from 0 to infinity, with values far removed from 1 indicating strong evidence in favor of one of the hypotheses, and values close to 1 indicating that the data do not strongly favor one hypothesis over the other. Such a continuous measure of support is exactly what is needed to be able to update one’s assessment of the plausibility of a hypothesis on the basis of new empirical evidence (Morey, Romeijn, & Rouder, 2016).

Before the procedure can be applied, the researcher has to decide on which hypotheses to consider. Since the procedure has not yet been extended to cover approximate null hypotheses, the focus will here be on evaluating the exact null hypothesis of equal variances, contrasting $H_{0}: \sigma ^{2}_{boys} = \sigma ^{2}_{girls}$ with its complement. She then needs to assess, based on her background knowledge, how plausible she takes both of these hypotheses to be. If she has strong background information that indicates that the variances are likely not equal while still not dismissing it entirely, she could specify *P*_*j*_(*H*_0_) = .1 and *P*_*j*_(¬*H*_0_) = .9. If she instead would have believed that there is no specific reason to suspect the assumption to be violated, it could be that choosing *P*_*j*_(*H*_0_) = .5 and *P*_*j*_(¬*H*_0_) = .5 had made sense to her (the default option offered by the procedure). The benefit of the procedure of Boeing-Messing et al. is that regardless of the choice of prior probabilities for the hypotheses, the Bayes factor that is obtained remains the same and provides an objective summary of the degree to which the data support one hypothesis over the other.

In this case, a Bayes factor of 1.070 is obtained, indicating that the data are almost equally likely to be observed under *H*_0_ as under ¬*H*_0_, and hence the data do not provide any real evidence in favor or against *H*_0_. Consequently, the posterior plausibility of *H*_0_ hardly differs from the prior plausibility: *P*_*j*_(*H*_0_|***X***) = .106 if our researcher used *P*_*j*_(*H*_0_) = .1.^5^ Thus, if she were skeptical about whether the model assumption held beforehand, she will remain skeptical about it after seeing the data. This is in stark contrast to the conclusion that she was led to using the confirmatory NHST approach, where the nonsignificant *p* value that was obtained led to an acceptance of *H*_0_. Thus, using this Bayesian approach in which she was able to take her background information into account, she has to conclude that there is little reason to accept that the model assumption holds, and has to question whether she should rely on inferences obtained based on models that assume equal variances. Developing more elaborate Bayesian procedures that allow for approximate hypotheses could be helpful here for assessing whether the assumption at least approximately holds. It may also be helpful for her to consider an estimation framework and attempt to assess the severity of the violation, as this could help her decide whether the model can still be relied upon to some degree.

## Conclusions

Evaluating the plausibility of model assumptions is a crucial step that needs to be passed before one can justify the use of a statistical model for making inferences. However, traditional NHST-based approaches to testing statistical model assumptions only provide ordinal information about the plausibility of the assumptions after having taken the data into account. Because of this, NHST-based procedures alone cannot provide sufficient information to determine whether it is plausible that the model assumptions hold and whether we can trust the inferences that are made using the model.

A good NHST-based test may still have the potential of providing a critical test for the model assumption, provided that the test is applied in the right situation: with high power and low *α*, obtaining a nonsignificant test statistic makes the model assumption much more plausible than it was before having observed the data. If the model assumption was already quite plausible to begin with, this might give us sufficient confidence in the assumption to apply the model. However, with low power or a priori implausible model assumptions, a nonsignificant result provides us with insufficient reason to accept the model assumption.

Because NHST does not take prior plausibility into account, it also cannot determine the actual power of the test and fails at providing the user with sufficient tools to adequately control the error rate that is most important when evaluating model assumptions: the probability of failing to reject an assumption that is violated. Thus, NHST-based approaches to evaluating model assumptions are insensitive to factors that should affect the conclusion that is drawn about the plausibility of the model assumption (see also Trafimow, 2003). Application of NHST without taking the prior plausibility into account may thus result in misleading conclusions about the plausibility of the model assumptions, which threatens the validity of the statistical inferences made using the model. Without taking this prior plausibility of model assumptions into account, it is unclear how we can be warranted in, for example, claiming that two groups differ significantly in their means, or that the mean difference is likely to fall within a certain interval, since all of these inferences depend on whether the model (at least approximately) holds.

By incorporating information about the prior plausibility of the model assumption and its alternatives in NHST-based testing procedures, the actual confirmatory power of such a procedure can be assessed. However, it may be more fruitful to abandon the idea that all the information about the model assumption is accurately captured by a dichotomized *p* value (see also Cohen, 1994; Wagenmakers, 2007), and make use of all the information that is available in the data to assess the assumption. Considering statistical measures such as the Bayes factor that capture the relative support of a model assumption over its complement may be more informative and relevant for determining the plausibility of that assumption (see for example Tijmstra, Sijtsma & Hoijtink, 2015).

Plausibility is not a dichotomous concept, even if in the end we do want to make a decision about whether the assumption is plausible enough to apply the model. Recognizing that there are degrees of plausibility and degrees of support is important, and we have to acknowledge that there are situations in which we may not be sure if we are confident enough about the truth of our model assumptions to use the model. If we conclude that the assumption is not as plausible as we would have liked, we will have to be more cautious in using the model to make inferences, or we may conclude that we do not have enough confidence in the model assumptions and refrain from applying the model, and have to search for a more general model that better captures the structure in the data. Estimating plausible sizes of suspected violations would also be helpful for determining the extent to which inferences can still be relied upon.

Prespecifying a general ‘minimal level’ of posterior plausibility that is needed before we can safely apply the model would ignore that different situations call for different degrees of certainty about our inferences. Confirmatory analyses may call for higher levels of certainty than exploratory analyses, as the evidential standards may be higher in the former case. High-stakes testing situations (e.g., for making decisions about individuals) may require even more certainty about the assumptions before we draw any conclusions. Thus, the choice for the required level of plausibility should depend on contextual factors.

Having to deal with prior plausibility may complicate the way in which model assumptions are evaluated, but it is at the gain of being able to determine how much confidence we should have in the statistical inferences that we make using our models. One can aim at minimizing possible subjective influences on this assessment of prior plausibility by formalizing explicit procedures for eliciting these epistemic judgments on the basis of the relevant information that is available (see for example O’Hagan et al., 2006), and by being explicit about the type of information that is considered to be relevant. The alternative—not assessing prior plausibility—is much more problematic, since under such an approach it will always remain uncertain whether a model can be trusted. Even worse, we will not have any information about the extent of this uncertainty. Thus, if we want our statistical and substantive conclusions to be valid, ignoring the prior plausibility of model assumptions is not an option.

## Footnotes

1 The results reported in this paragraph have been obtained based on a set of fictional data, created for illustrative purposes and available in the supplementary material of this manuscript on the Open Science Framework at http://osf.io/rh8qn.

2 With the notable exception that in practice approximate null hypotheses are likely to be much more plausible.

3 It may be noted that calculating the power based on the estimated effect size (i.e., post hoc power analysis) does not avoid this issue, since it amounts to assuming prior knowledge that each possible alternative to *H*_0_ is equally probable (e.g., see Jeffreys, 1961, pp. 117/118), which often may not be a reasonable starting point.

4 Since we are considering a significance testing framework, it is assumed here that we are only conditioning on whether a significant result was obtained, and not on the exact *p* value.

5 And *P*_*j*_(*H*_0_|***X***) = .517 if she had used *P*_*j*_(*H*_0_) = .5.

## References

[CR1] Berger JO, Delampady M (1987). Testing precise hypotheses. Statistical Science.

[CR2] Berger JO, Sellke T (1987). Testing a point null hypothesis: The irreconcilability of P values and evidence. Journal of the American Statistical Association.

[CR3] Böing-Messing F, van Assen MA, Hofman AD, Hoijtink H, Mulder J (2017). Bayesian evaluation of constrained hypotheses on variances of multiple independent groups. Psychological Methods.

[CR4] Box GEP, Draper NR (1987). Empirical model-building and response surfaces.

[CR5] Cohen J (1994). The earth is round (p < 0.05). American Psychologist.

[CR6] Cumming G (2014). The new statistics why and how. Psychological Science.

[CR7] Cumming G, Finch S (2005). Inference by eye: Confidence intervals and how to read pictures of data. American Psychologist.

[CR8] Earman J (1992). Bayes or bust.

[CR9] Edwards W, Lindman H, Savage LJ (1963). Bayesian statistical inference for psychological research. Psychological Review.

[CR10] Fidler F, Loftus G (2009). Why figures with error bars should replace p values: Some conceptual arguments and empirical demonstrations. Zeitschrift für Psychologie/Journal of Psychology.

[CR11] Field A (2009). Discovering statistics using SPSS.

[CR12] Fisher RA (1930). Statistical methods for research workers.

[CR13] Fisher RA (1955). Statistical methods and scientific induction. Journal of the Royal Statistical Society (B).

[CR14] Fisher RA (1956). Statistical methods and scientific inference.

[CR15] Fisher RA (1960). The design of experiments.

[CR16] Fitelson B (2006). Logical foundations of evidential support. Philosophy of Science.

[CR17] Fitelson B (2007). Likelihoodism, Bayesianism, and relational confirmation. Synthese.

[CR18] Gigerenzer, G. (1993). The superego, the ego, and the id in statistical reasoning. In G. Keren, & C. Lewis (Eds.) *A handbook for data analysis in the behavioral sciences: Methodological issues* (pp. 311–339). Hillsdale: Erlbaum.

[CR19] Gigerenzer G (2000). Adaptive thinking: Rationality in the real world.

[CR20] Gigerenzer G (2004). Mindless statistics. The Journal of Socio-Economics.

[CR21] Gigerenzer G, Murray DJ (1987). Cognition as intuitive statistics.

[CR22] Gigerenzer G, Swijtink Z, Porter T, Daston L, Beatty J, Krüger L (1989). The empire of chance. How probability changed science and every day life.

[CR23] Gillies D (2000). Philosophical theories of probability.

[CR24] Guilford JP (1978). Fundamental statistics in psychology and education.

[CR25] Hacking I (1976). Logic of statistical inference.

[CR26] Hoijtink HJA (2012). Informative hypotheses: Theory and practice for behavioral and social scientists.

[CR27] Howson C, Urbach P (1989). Scientific reasoning: The Bayesian approach.

[CR28] Hubbard R, Bayarri MJ (2003). Confusion over measures of evidence (p’s) versus errors (α’s) in classical statistical testing. The American Statistician.

[CR29] Jaynes ET (1968). Prior probabilities. IEEE Transactions on Systems Science and Cybernetics.

[CR30] Jaynes ET (2003). Probability theory: The logic of science.

[CR31] Jeffreys H (1961). Theory of probability.

[CR32] Kadane JB, Wolfson LJ (1998). Experiences in elicitation. The Statistician.

[CR33] Kass RE, Raftery AE (1995). Bayes factors. Journal of the American Statistical Association.

[CR34] Lee MD, Wagenmakers E-J (2005). Bayesian statistical inference in psychology: Comment on Trafimow (2003). Psychological Review.

[CR35] Lehmann EL (2006). The Fisher, Neyman–Pearson theories of hypothesis testing: One theory or two?. Journal of the American Statistical Association.

[CR36] Levene, H. (1960). Robust tests for equality of variances. In I. Olkin, S. G. Ghurye, W. Hoeffding, W. G. Madow, & H. B. Mann (Eds.) *Contributions to probability and statistics: Essays in honor of Harold Hotelling* (pp. 278–292). Stanford University Press: Stanford.

[CR37] Lord FM (1980). Application of item response theory to practical testing problems.

[CR38] Mayo DG (1996). Error and the growth of experimental knowledge.

[CR39] Meehl PE (1978). Theoretical risks and tabular asterisks: Sir Karl, Sir Ronald, and the slow progress of soft psychology. Journal of Consulting and Clinical Psychology.

[CR40] Morey RD, Romeijn JW, Rouder JN (2016). The philosophy of Bayes factors and the quantification of statistical evidence. Journal of Mathematical Psychology.

[CR41] Neyman J (1937). Outline of a theory of statistical estimation based on the classical theory of probability. Philosophical Transactions of the Royal Society, Ser A.

[CR42] Neyman J (1957). Inductive behavior as a basic concept of philosophy of science. International Statistical Review.

[CR43] Neyman J, Pearson ES (1967). Joint statistical papers.

[CR44] Nickerson RS (2000). Null hypothesis statistical testing: a review of an old and continuing controversy. Psychological Methods.

[CR45] O’Hagan A (1998). Eliciting expert beliefs in substantial practical applications. The Statistician.

[CR46] O’Hagan A, Buck CE, Daneshkhah A, Eiser JR, Garthwaite PH, Jenkinson DJ, Rakow T, ... (2006). Uncertain judgements: Eliciting experts’ probabilities.

[CR47] Pearson ES (1955). Statistical concepts in their relation to reality. Journal of the Royal Statistical Society (B).

[CR48] Popper KR (1959). The logic of scientific discovery.

[CR49] Ramsey, F. P. (1926). Truth and probability. In *Ramsey 1931*. Reprinted in H. E. Kyburg and H. E. Smokler (eds.), *Studies in subjective probability*, 1964, 61–92 (pp. 156–198). New York: Wiley.

[CR50] Savage LJ (1972). The foundations of statistics.

[CR51] Student (1908). The probable error of a mean. Biometrika.

[CR52] Suppes P (2007). Where do Bayesian priors come from?. Synthese.

[CR53] Tabachnick BG, Fidell LS (2001). Using multivariate statistics.

[CR54] Tijmstra J, Sijtsma K, Hoijtink HJA (2015). Evaluating manifest monotonicity using Bayes factors. Psychometrika.

[CR55] Trafimow D (2003). Hypothesis testing and theory evaluation at the boundaries: Surprising insights from Bayes’s theorem. Psychological Review.

[CR56] Wagenmakers E-J (2007). A practical solution to the pervasive problem of p values. Psychonomic Bulletin & Review.

[CR57] Wagenmakers E-J, Grünwald P (2005). A Bayesian perspective on hypothesis testing. Psychological Science.

[CR58] Wasserstein RL, Lazar NA (2016). The ASA’s statement on p-values: Context, process, and purpose. The American Statistician.

[CR59] Welch BL (1947). The generalization of ‘Student’s’ problem when several different population variances are involved. Biometrika.

